# Understanding the impacts of the COVID-19 response measures on Deaf adults in Cape Town

**DOI:** 10.4102/ajod.v13i0.1371

**Published:** 2024-06-19

**Authors:** Charlotte Slome, Myrna van Pinxteren, Leslie London

**Affiliations:** 1 School of Public Health and Family Medicine, Faculty of Health Sciences, University of Cape Town, Cape Town, South Africa; 2 Division of Social and Behavioural Sciences, Faculty of Health Sciences, University of Cape Town, Cape Town, South Africa

**Keywords:** deaf, COVID-19, information access, South Africa, access to healthcare

## Abstract

**Background:**

International literature has evidenced that Deaf people have been disadvantaged during the COVID-19 pandemic; however, there is currently little research published within the South African context.

**Objectives:**

This study investigated the ways in which the COVID-19 pandemic and its consequent response measures impacted Deaf adults in Cape Town.

**Method:**

Using a descriptive approach, semi-structured, qualitative interviews were held with 15 Deaf adults in Cape Town, South Africa. Participants were purposively selected through a local Deaf organisation. Data were analysed using thematic analysis.

**Results:**

Data revealed the challenges experienced when accessing information, the impact of communication barriers on daily life, and how the response measures impacted access to healthcare.

**Conclusion:**

The findings of this study demonstrate how the needs of the Deaf community were overlooked and their voices disregarded during the planning of the national pandemic response, ultimately having detrimental consequences. Therefore, the authors argue for greater inclusion of Deaf representatives to ensure equal access to information and resources, especially during a crisis.

**Contribution:**

This study contributes to the growing body of knowledge on the consequences of the COVID-19 pandemic in the field of disability and insights can inform both future research and interventions to promote equity and inclusion for Deaf people.

## Introduction

### The COVID-19 pandemic for persons with disabilities

The COVID-19 pandemic, caused by severe acute respiratory syndrome coronavirus 2 (SARS-CoV-2), disrupted and changed lives worldwide. Socially disadvantaged populations, including persons with disabilities (PWDs) suffered considerably, not only from the direct impacts of the virus, but also from the unintended economic and social consequences of response measures implemented to control the pandemic (Gashaw, Hagos & Sisay 2021). Global data has shown that in many disaster and emergency situations, the mortality rate of the population of PWDs is two to four times higher than that of the population of persons without disabilities, more so due to discriminatory policies and practices than the disability itself (Stough & Kelman 2018). In the context of the COVID-19 pandemic, PWDs have more than twice the risk of dying compared to persons without disabilities (Kuper & Smythe 2023). Persons with disabilities have reported being excluded from economic interventions, such as emergency cash transfers, access to routine healthcare and access to information (Banks et al. 2020). These consequences are often worse in low- and middle-income countries (LMICs) as circumstances are compounded by existing poverty, strained health systems and disease burden (Engelbrecht et al. 2023). Although there are some parallels between Deaf experiences compared to other disabilities, the centrality of language in the Deaf experience is often overlooked in wider disability studies (Kusters 2011).

### Deaf persons’ pandemic experiences

The prevalence of disabling hearing loss is estimated to be 5% of the global population, and nearly 80% living in LMICs (World Health Organization 2024). Deafness is a spectrum and encompasses a wide array of experiences (Enright 2022). The term ‘deaf’ denotes the medical model of deafness as a pathology and includes any person whose hearing loss is greater than 35 decibels (dB), while those with some residual hearing are considered hard-of-hearing (Hoh) (WHO 2024). Whereas the term ‘Deaf’ refers to deaf or hard-of-hearing people who primarily use a signed language and identify as members of the Deaf community, a cultural-linguistic group that shares common values, norms, behaviours, traditions and language (Heap & Morgans 2006). Signed languages have their own syntax, semantics and pragmatics, which are different from their spoken counterparts and other signed languages. For example, American Sign Language (ASL) is not the direct translation of English and bears little similarity to British Sign Language.

A majority of deaf babies (90%) are born to hearing parents who have little to no understanding of deafness or Deaf culture and do not know the relevant signed language, which often hinders the child’s linguistic development and learning (Chininthorn et al. 2016). Systemic barriers and discrimination against Deaf people have resulted in pervasive, multidimensional inequalities. Compared to those who can hear, Deaf people globally experience high rates of poverty, unemployment, and low literacy and education levels (Barnett et al. 2011; Chininthorn et al. 2016) – all of which impact their experience within the health system and their overall health and well-being. Several international studies have provided evidence that the pandemic-related circumstances have amplified and exacerbated existing inequalities for Deaf people, including communication, access to information and access to healthcare (Park 2020; Swanwick et al. 2020).

### The South African Deaf community

The prevalence of Deaf South African Sign Language (SASL) users in South Africa is difficult to ascertain, as there are no updated statistics nor reliable measurement instruments, but historical estimates range between 500 000 and 1.5 million, or 0.84% – 2.5% of the population (London, Zweigenthal & Heap 2020). Due to educational barriers, the average reading and writing level among Deaf school leavers is at a Grade 4 equivalent, resulting in 75% of South African Deaf adults functionally illiterate and 70% of the Deaf population are unemployed (Chininthorn et al. 2016). Language and communication barriers, mistreatment from staff and nonadherence to medication are commonly reported by Deaf patients accessing the South African health system (Gichane et al. 2017; Kritzinger et al. 2014).

Unlike most other groups, the Deaf community is a population without a geographic base, making Deaf organisations a central node in their social networks (Heap 2003). One such organisation is the Deaf Community of Cape Town (DCCT). Founded in 1987 as a non-governmental organisation (NGO) by Deaf people, for Deaf people, DCCT works to address the needs of the Deaf community in the Western Cape province. Members come to DCCT for counselling services, booking interpreters, employment opportunities, skill-building and social engagements. The efforts of disabled persons’ organisations (DPOs) during the pandemic to support their communities and contribute to research, despite a lack of support from government have been documented globally (Hillgrove et al. 2021; Hlongwane et al. 2022).

### South Africa’s COVID-19 response

Despite the United Nations (UN) Disability-Inclusive Response to COVID-19 report emphasising that PWDs be included in planning COVID-19 responses, the South African government did not meaningfully engage PWDs at any stage of the response (Mulibana 2020). Shortly after the World Health Organization (WHO) declared it a global pandemic, South Africa announced a national State of Disaster on 15 March 2020, under which a five-level alert system was implemented to place degrees of restrictions on the freedoms of movement and assembly. Although cases were relatively low at the time and no deaths had been recorded, the measures were deemed necessary to curb the spread of the virus, given the population’s existing vulnerabilities in terms of the high disease burdens of human immunodeficiency virus (HIV) and tuberculosis (TB), high levels of poverty, high urban population density and resource-limited public health system (Ikwegbue et al. 2021). Just 9 days after the first locally transmitted case was detected, a shelter-in-place order was implemented under the Level 5 lockdown, requiring all citizens to stay at home except for certain permitted activities, such as obtaining essential goods or services, seeking emergency or chronic medical attention, or collecting a social grant (South African Government 2020). Additionally, public health measures including hand hygiene, compulsory wearing of a mask in public spaces and social distancing were implemented (South African Government 2020).

This led to discriminatory practices reported by PWDs during the pandemic, including the inability to access government stimulus packages, food parcels and social grants (Ned et al. 2020). Persons with disabilities also reported challenges accessing their medications, attending appointments without support personnel or reliable transportation, and inadequate communication regarding the rescheduling or cancellation of routine services (Ned et al. 2020). South African Deaf adults expressed challenges accessing information during the pandemic without regular, good quality interpreters (Adigun, Vivekanantharase, & Obosu 2021).

While there is some evidence of the impacts of the COVID-19 lockdown on Deaf people in other countries, there is little research published examining the experiences and impacts of COVID-19 on Deaf people in South Africa. The aim of this study was to assess the effect of the response measures on Deaf persons’ access to pandemic-related information, ability to communicate, social functioning and access to health and social services.

## Research methods and design

### Research design and setting

This study employed a qualitative data collection method, using a descriptive design to explore the experiences of Deaf persons during the COVID-19 pandemic. A descriptive design is especially suitable in research areas where little is known, allowing the researcher to expose the subjective experiences and perceptions of the participants (Bradshaw, Atkinson & Doody 2017). There are two Deaf organisations in Cape Town, namely, the Deaf Federation of South Africa (DEAFSA) and DCCT. The DCCT was chosen as an appropriate research site due to its long-standing relationship with members of the research team and previous experience with research. Deaf Community of Cape Town is located in a Cape Town suburb of mixed middle and low socioeconomic status. Many of the roughly 1000 beneficiaries travel from all across metropolitan Cape Town to attend DCCT.

### Study population and sampling

The target population was Deaf adults in Cape Town. A purposive sampling method with maximum variation was used to recruit participants, allowing the researcher to generate a sample from the targeted population that was both accessible and met the inclusion criteria (Ulin, Robinson & Tolley 2005). Due to COVID-19 regulations restricting the researcher’s access to potential participants, a DCCT staff member with experience in research assisted in recruiting participants who represented a wide variety of social and individual characteristics. For inclusion, participants had to be over 18 years, residing in Cape Town, be deaf or Hoh, identify as part of the Deaf community, communicate primarily in SASL, and agree to comply with COVID-19 safety precautions. Originally, those above 60 were considered high-risk for COVID-19 infection and thus, were excluded from the study. However, during recruitment, DCCT held an event for the elderly and the study recruiter selected a few to be participants. Given that these individuals were already attending DCCT, this study posed no additional COVID-19-related threat and as such, they were included. In line with Saunders et al. (2018), fieldwork was completed once no additional new data were introduced by participants. In our study, this occurred after the 15th interview.

### Data collection

Data were collected through semi-structured, in-depth interviews. Interviews were conducted in a private room at the DCCT centre. The interview site met both SASL-specific requirements (e.g. good lighting, low background noise and distractions) and COVID-19 precautions, based on national guidelines. The interview schedule was developed after thorough review of global literature on the impacts of the pandemic on Deaf people, extensive discussions among the research team and input from Equal Health for Deaf People (EH4DP n.d.). The EH4DP is a non-profit organisation (NPO), which works with the Deaf community to eliminate language barriers, promote Deaf awareness in the health system and ultimately, advance the right to health for all Deaf people. Based on feedback from the two pilot interviewees and interpreters, the interview guide was shortened slightly to ensure questions remained relevant to the subject and participants would not be fatigued. Pilot interviews were not included for the main study.

During the interview, participants were asked to describe their experience of the pandemic and its response measures, the impact on their ability to communicate and access services and their ability to access COVID-19-related information. Interviews were conducted in English and SASL by the first author with the use of two professional SASL interpreters, certified through the South African Translators Institute (SATI) and employed by the University of Cape Town (UCT). The first author would ask questions in English, which were translated by the interpreter into SASL, allowing the participant to respond in SASL, and those responses were then translated back into English by the interpreter. The first author has a background of Deaf studies, is proficient in ASL, and has experience using interpreters. Both interpreters are trained in research ethics, have prior experience interpreting for research, and were briefed about the study prior to starting. Interviews were on average 30 min and the researcher worked with one interpreter per interview, as the content and duration of interviews were not deemed as needing more than one interpreter to avoid fatigue. Each session was videotaped in order to capture the original signed data, with one frame on the interviewee and another frame on the interpreter. Observational and reflective fieldnotes were generated as a complementary method of data collection. Interview recordings were transcribed from SASL into written English before analysis commenced.

### Data analysis

The textual data were analysed using thematic analysis. Transcriptions and fieldnotes were reviewed iteratively and thematically coded and stored in NVivo. The data analysis process followed the six steps of thematic analysis as defined by Braun and Clarke (2012):

- Familiarising yourself with the data;
- generating initial codes;
- searching for themes;
- reviewing themes;
- defining and naming themes;
- producing the report.

The initial round of coding was done deductively, where themes related to the study objectives were applied to the data to generate codes. A subsequent, inductive process allowed codes and themes to emerge from the content of the data. Several rounds of consultation with co-authors occurred to discuss the generated themes and interpretations and were reviewed with a DCCT representative and interpreters to gain additional context and ensure content validity. Recurring themes were then grouped together until the four final themes emerged, which reflected the disruptions to their social and economic lives, experiences accessing COVID-19 related information, challenges with interpersonal communication and their experiences in the health system.

To promote rigour of the findings, the researchers adopted Lincoln and Guba’s (1985) four criteria. Fieldnotes were used as a method of data triangulation to promote the credibility of the data and depth of the findings. Although the findings cannot be transferable to other contexts, the authors provided a rich description of the study context and methodological steps. An audit trail of the raw data, fieldnotes and transcripts was kept to ensure dependability. Lastly, a detailed account of the production of codes, themes and findings as well as a reflective journal of the first author’s thoughts and experiences were kept to address confirmability.

### Ethical considerations

The consent form was previously used in studies conducted with the Cape Town Deaf community and was piloted and adjusted based on participant feedback. The consent process was videotaped to record participants’ decision as a signature, in order to capture the original data. Participants were given a copy of the information sheet in local languages: English, isiXhosa, or Afrikaans. Interpreters signed a confidentiality agreement, stating they would not discuss nor share research information with anyone outside the research team. Ethical clearance to conduct the study was obtained from the University of Cape Town’s Human Rights and Ethics Committee (HREC REF: 198/2021).

## Results

Fifteen interviews were conducted between September and October 2021. Participants’ ages ranged between 26 and 67 years. Ten participants identified as women, five as men. Six were unemployed at the time of interviews, while nine were employed. In terms of the highest level of education achieved, nine participants had below or were at a Grade 8 level, six had between Grades 9 and 12. All participants were culturally Deaf and considered SASL as their primary language. Nine lived only with hearing people who had basic or no SASL knowledge, one lived alone, and five lived with at least one other fluent SASL user. Participant characteristics are displayed in Table 1. For example, Participant 5 is a 41-year-old Deaf man, who is unemployed, and lives with at least one other hard-of-hearing person who is fluent in SASL and at least one other hearing person who knows only basic SASL. We have included participants’ home environment as this impacts their level of knowledge, access to information and social isolation, all of which are important findings of this paper.

**TABLE 1:** Participant characteristics.

Participant number	Sex	Age (years)	Educational attainment	Employment status	Home environment
Participant 1	F	60	Grade 8	Unemployed	Hearing (basic)
Participant 2	F	67	Grade 8	Unemployed	Alone
Participant 3	F	38	Grade 11	Employed	Hearing (basic)
Participant 4	F	63	Grade 6	Unemployed	Hearing (basic)
Participant 5	M	41	Grade 8	Unemployed	Hoh (fluent); Hearing (basic)
Participant 6	F	26	Grade 9	Unemployed	Hearing (basic)
Participant 7	M	30	Grade 10	Employed	Hearing (basic)
Participant 8	F	45	Grade 12	Employed	Hearing (basic)
Participant 9	F	26	Grade 9	Employed	Hoh (fluent)
Participant 10	M	45	Grade 7	Employed	Hoh (fluent); Hearing (basic)
Participant 11	F	47	Grade 8	Employed	Deaf (fluent); Hearing (basic)
Participant 12	F	55	Grade 9	Employed	Hearing (fluent); Hearing (basic)
Participant 13	F	58	Grade 4	Employed	Hearing (basic)
Participant 14	M	47	Grade 5	Unemployed	Hearing (basic)
Participant 15	M	45	Grade 8	Employed	Hearing (none)

F, female; M, male; Hoh, hard-of-hearing.

Four relevant themes were derived from the data analysis:

1. disruption of social and economic life of Deaf people during COVID-19,
2. information provision during the COVID-19 pandemic,
3. the impact of the response measures on interpersonal communication and
4. the impact of the response measures on access to health care. Each theme is discussed below.

### Disruption of social and economic life of Deaf people during COVID-19

The COVID-19 response measures, specifically the lockdown, had a devastating socio-economic impact on participants. Three participants lost their jobs due to business closures. The sudden loss of income caused financial strain on participants and their families. The stress of having to choose between budgeting for food and other necessities or masks and sanitiser was a common sentiment:

‘I worked at a hotel, but they closed in March 2020 and sent us all home. The managers asked us to write down our contact information and said they would call, but six months passed, and they never called. They finally called my mom in March 2021 to facilitate the Unemployment Insurance Fund (UIF) process. During that time, I had no income.’ (Participant 14, Male, 47years old)

Aside from financial difficulties, participants also lost contact with their Deaf peers. Nine participants (60%) lived with only hearing family members not proficient in SASL, which was particularly difficult during the shelter-in-place period, as they lost contact with their signing community and felt socially isolated at home. Participants who had access to a smartphone or laptop were able to video chat with their friends to stay in touch, but this was limited by the high cost of data:

‘We would have video calls but sometimes you don’t have money for data and then you had to miss out for that.’ (Participant 8, Female, 45 years old)

Others described how the lockdown disrupted their daily lives and social support systems, the fear around the severity of the disease itself and the grief from losing loved ones:

‘I was sad because a lot of people I know passed away from COVID. One person from my church contracted COVID, which was an overwhelming situation because I had to self-isolate and stay home. It felt like I was in prison.’ (Participant 1, Female, 60 years old)

One participant expressed how she did not feel terribly affected by the lockdown regulations because they did not drastically conflict with her regular living habits:

‘I don’t know. I’m able to sanitize and adhere to the rules. So, I haven’t been infected or affected by it. During lockdown it was like no worries for me because I don’t drink or smoke. Just tea or coffee, that’s my thing.’ (Participant 2, Female, 67 years old)

### Information provision during the COVID-19 pandemic

The lack of accessible information was a serious concern among respondents. All participants had general knowledge of COVID-19 measures such as social distancing, hand hygiene and masking. However, reflecting on their experiences during shelter-in-place restrictions at the beginning of the pandemic in early 2020, they felt extremely uninformed. Participants were asked to share the sources they relied upon for COVID-19-related information. Fourteen participants (93%) identified DCCT as a main source of information.

Accessing information was especially difficult during shelter-in-place orders as the structures they generally relied on for social interaction and information, such as DCCT, were closed. Only three participants mentioned getting some information from their hearing family, but this information was hard to digest as their family members were not proficient in SASL. Participant 7 reflected on his experience accessing information while living at home during the lockdown:

‘My brothers sign a little, but it isn’t much. They try to explain what the news is saying. I could have full communication with my late mother, but my brothers sign very slow. But I have to accept the situation.’ (Participant 7, Male, 30 years old)

The televised interpreted news was the second most common source of information, as identified by 10 participants. TV channels had an interpreter every day at 17:30 and 20:00. However, participants expressed frustration regarding the inconsistency and inadequacy of the interpreted news, stating it was not enough to provide full access to information:

‘30 minutes only? That’s not enough, that’s not the details. On SABC1 we would be missing interpreters. During lockdown the interpreter wasn’t on at night. Hearing people can access the news any time, but for Deaf people, our time is limited. They need to have more interpreters on the news channels.’ (Participant 8, Female, 45 years old)

The lack of access to information was also apparent in their daily conversations and social interactions. A participant who worked with other Deaf adults described how they had to fight for the same information that was provided to their hearing co-workers:

‘My workplace explained about COVID but not with an interpreter and using basic signs, so we didn’t understand a lot. When we asked what the manager said, they would say “you must wait … you must wait.” But it’s important, I need to know now!’ (Participant 3, Female, 38 years old)

This exclusion from information and lack of context or detail provided by hearing people was a common experience and elicited feelings of anger and disappointment:

‘When Deaf people ask, “what does that mean,” they’ll tell you one word and that’s all the information they share. You can see the person is sharing a lot of information but when you ask, they give you one or two words.’ (Participant 8, Female, 45 years old)

The lack of access to information also led to Deaf people not following social distancing or masking rules. Several participants reported not masking or social distancing during the initial stages of the pandemic, thus increasing their risk of COVID-19 infection. Participant 4 shared that in the beginning of the lockdown she did not adhere to imposed restrictions because she did not realise the threat that COVID-19 posed:

‘But I said, “what is dangerous?” I was told to stay home for 6 weeks but I’d go to visit my friends because I didn’t know what is this Corona. What does it mean? I didn’t really understand anything … I had no information while I was staying at home during lockdown levels 4 and 5.’ (Participant 4, Female, 63 years old)

She attributed her behaviour to being ill-informed of the restrictions and the importance of adherence as a means of protection.

Deaf Community of Cape Town played a pivotal role in addressing the information gaps throughout the pandemic. However, as DCCT was not considered an essential service during the initial lockdown restrictions, the organisation had to close its doors from March to June 2020. During this time, DCCT created COVID-19 awareness videos in SASL, using information from the televised presidential addresses and the Department of Health’s website. These videos were disseminated through Facebook and WhatsApp, and participants referred to these videos as their main source of information. One participant emphasised the importance of DCCT’s role in information provision, given the inaccessibility of other information sources:

‘There are many Deaf people who only understand what’s happening because of the information DCCT shares, because Deaf people don’t have equal access to the same information as hearing people.’ (Participant 15, Male, 45 years old)

After participants received information in SASL, they understood both the severity of the disease itself, and how the response measures could protect them from infection, and thus, began to comply with lockdown rules. Participant 4 recalled how she had to wait 3 months, until June 2020, when DCCT was allowed to open under Level 3 restrictions, to receive any intelligible information about COVID-19 and the pandemic response measures:

‘After a long time, I came to DCCT and they shared information in SASL and I was like, “Oh, it’s Corona, it’s dangerous, it’s an infection.” So, I say thank you to DCCT because it was only when they started sharing information that I understood. After I understood, I became very strict and careful.’ (Participant 4, Female, 63 years old)

When restrictions began to ease and in-person gatherings were permitted, DCCT held events both at the centre and within communities to further promote education around COVID-19:

‘DCCT would travel and have information sessions to explain why we must socially distance, sanitise, and mask. They also gave us bags with masks and sanitisers because they said they don’t want Deaf people to die, we want to protect you. Then I started to really believe.’ (Participant 4, Female, 63 years old)

Similar to before the pandemic, DCCT served as a hub for the Deaf community, where they could come together to support one another and share information. This especially benefited DCCT employees, as they were able to regularly share and discuss COVID-19-related information. One employee described the process of information sharing among staff and how such information was further disseminated into the community:

‘Staff members would get the information and then do a presentation teaching us about Corona and how to protect ourselves. Basically, it was a training session for us so we could then share information with other Deaf people.’ (Participant 8, Female, 45 years old)

Participants also expressed how important it was for them to be able to support their Deaf peers, recognising how difficult it can be for some to access information:

‘If you don’t understand anything about COVID and I have some information, I love to share. Mostly to help Deaf people because it’s very difficult to understand what COVID is. They just thought it’s not safe, but knowing in detail what it entails and how it spreads, this is what we need as Deaf people, to be aware and have more information about COVID.’ (Participant 6, Female, 26 years old)

Participants described the inaccessibility of COVID-19 information and the subsequent information deficit, which influenced their behaviours and attitudes towards the response measures. In the face of such barriers to accessing information, participants also revealed the strategies they adopted to overcome and navigate the lack of information. The organised actions of DCCT were crucial in providing accurate and understandable COVID-19-related information and support to the Deaf community. Information sharing on an individual level was also commonly discussed, and participants described the personal responsibility they felt to share the information they had with their Deaf peers.

### The impact of the response measures on interpersonal communication

Aside from participants feeling uninformed by the lack of access to timely COVID-19 information, they were also challenged in interpersonal communication, with both hearing and Deaf people. While communicating with hearing non-signers has always been challenging, the response measures, and masking in particular, exacerbated existing communication barriers. Most participants relied on lip-reading when communicating with hearing people prior to the pandemic and although this communication mode is not ideal, as it is mentally taxing and only moderately effective, masking completely thwarted the ability to lip-read. When participants asked hearing people to remove their mask or for clarification of what was said, they were met with hostile reactions. Due to the communication barriers created by masking, participants had to resort to written communication, which presented its own challenges. Grammatical differences between SASL and the local languages (English, isiXhosa and Afrikaans) often made it difficult to discern what was written. Additionally, due to social distancing mandates and the fear of infection, participants described how many hearing people were hesitant to share pen and paper or get close enough to read their notes:

‘If I ask people to remove their mask, they refuse. So, I have to write things, but some would get angry. They’d say, “What does this mean, what is this about?” Sometimes you just give up and have to go to the next person until you find someone who’s understanding.’ (Participant 13, Female, 38 years old)

The communication barriers caused by masking, social distancing and lockdown regulations also disrupted daily activities such as taking taxis, going to the bank or dealing with social services like the South African Social Security Agency (SASSA) or police services:

‘I brought an interpreter with me to the SASSA office, but they said the interpreter must keep their mask on. We explained that we need to see each other’s faces for facial expressions, but they refused. We tried to communicate with the mask on, but it was quite difficult. Some places, like SASSA, had their own rules about the mask so we just had to adhere to their rules.’ (Participant 5, Male, 41 years old)

These COVID-19 mandates are often enforced by security personnel employed by SASSA and police services, who are not trained to accommodate people with different needs. One participant described how masking-related communication barriers led her to get into a shared minibus taxi travelling to the wrong suburb in Cape Town, having both time and financial consequences:

‘It was very difficult and challenging to communicate. When you’re asking a taxi, you have to write down everything. “Where are you going;” “I’m going to Cape Town.” Sometimes people would tell me the wrong taxi to get on. And that taxi goes to Sea Point and not Cape Town. So that was very stressful for me because of the masks.’ (Participant 8, Female, 45 years old)

While communication barriers with hearing people existed prior to the pandemic, masking created a novel challenge for Deaf people when communicating in SASL. Participants described how it was difficult to identify emotion and other aspects of grammar, conveyed through facial expressions in signed languages, when a large portion of one’s face is hidden behind a mask, resulting in frequent miscommunications:

‘You must take off your mask when signing with other Deaf people. They need to see your lips and mouth. It’s part of the language. If you cover your mouth, they get closer to you and think you’re angry because the mask is covering your face and then you have to take it off and say, “[*N*]o, I’m not angry”.’ (Participant 11, Female, 47 years old)

Having to choose between full communication and protecting themselves from infection placed many in a difficult predicament. Participants also noted that social distancing created a communication barrier because it goes against Deaf culture and customs. While it is easy to use one’s voice to get the attention of a hearing person who may be facing the other way or not paying attention, to get the attention of a Deaf person often requires tapping them on the shoulder, flickering the lights or moving around to the front of them, some of which could break social distancing rules. One participant captured this dilemma:

‘It was difficult. We must touch each other because we are Deaf. How am I going to call a Deaf person if they’re focusing on something else and don’t see me waving my hands? So, I have to tap them.’ (Participant 6, Female, 26 years old)

Several others described how social distancing also inhibited the important practice of physical touch in Deaf culture:

‘Social distancing is difficult for Deaf people. Deaf people want to sit next to you, be close. I would always have something between us to make social distancing but then we can sign to each other. Because we are a close community, it’s part of Deaf Culture. It’s the Deaf way.’ (Participant 4, Female, 63 years old)

### Practical barriers of the COVID-19 response measures on access to health services

Nearly all participants (*n* = 14) accessed health services during the pandemic. During the initial stages of South Africa’s lockdown, interpreting services were not deemed essential and thus, interpreters were not allowed to accompany Deaf patients. Among the 14 participants who accessed health services during the pandemic, 10 (71%) expressed a desire to have an interpreter but were unable to do so due to the restrictions. A participant described her experiences being denied an interpreter when accessing care:

‘When the ambulance came to take my husband to the hospital because he had COVID, I asked if my son could go with him because he’s hearing and my husband is Hoh, but she said no, he needs to go alone. This was in January 2021.’ (Participant 12, Female, 55 years old)

In the few days before her husband passed away in hospital, there was a lack of communication from health workers and the little information shared was relayed through her hearing son, which made her feel excluded and isolated.

Among the three participants who were allowed to bring someone to interpret, only one was able to have a professional interpreter. The other two were only allowed to bring a family member. Participant 14 explained that his mother always attended his monthly appointments to assist with communication, so clinic staff made an exception by allowing her to accompany him in spite of pandemic regulations:

‘There was no interpreter, so my mom went with me. The doctor would’ve sent me back because we struggle to communicate so my mom must come with. I have a monthly appointment and they know I’m Deaf so they would call me, but I can’t hear, so my mom always comes to help me.’ (Participant 14, Male, 47 years old)

Participant 3 described the challenge of having to rely on her daughter, who is not fluent in SASL, to attempt to bridge the language gap between herself and healthcare providers:

‘My daughter told them, “My mom is Deaf and I’m the daughter” and they allowed her to come. She did try. It was a slow process to get information across, but we finally managed, and I got my medication.’ (Participant 3, Female, 38 years old)

As interpreters were not allowed at the clinic, some participants had negative experiences with healthcare workers who refused to make accommodations to facilitate communication. Being unable to lip-read because of masks made it impossible for patients in the waiting room to see their name being called for their appointment. One participant detailed his experience:

‘I saw they were calling patient’s names, but I couldn’t see if mine had been called because they were wearing a mask. I wrote a note, but they said, “No sorry, we can’t hear you.” I asked them to please speak more clearly but the receptionist just started pointing. I was like, “What does that mean? How are you communicating with me like that? I need you to put down your mask so I can communicate or write things down,” but they refused. There was a window too. “If there’s a window, why wouldn’t you pull down your mask?” They said no and ignored me.’ (Participant 15, Male, 45 years old)

Written communication was used most commonly during consultations, as providers refused to remove their masks. Among those who used this method, the majority were dissatisfied with the experience:

‘Now with masks everywhere, I have to ask people to please write. But sometimes they would say, “What are you writing here, what is this? I don’t understand.” It would make me feel uneasy. Because of COVID this is my only option, you need to at least accept this.’ (Participant 6, Female, 26 years old)

Not fully understanding the prescribed treatment because of providers’ limited ability to communicate was also a common experience, which led to feelings of fear and frustration as well as defaulting from medication:

‘The doctor changed my blood pressure tablets during Level 4, but the nurses wouldn’t let me see the doctor and didn’t explain why they changed medication. They just brought the medication and told me to go home. The new tablets made me very dizzy so when I finally saw the doctor during Level 3, I told them I don’t want to take this medication because I don’t know what it’s for and I’m afraid.’ (Participant 11, Female, 47 years old)

There were several instances where participants devised creative strategies to address these barriers. To circumvent issues of being unable to see when the nurses call for your appointment due to masks, one participant decided to write ‘Deaf’ on his medical folder as an alternative method for staff to communicate when it was time for his appointment:

‘The nurses think, “[*H*]ow am I going to communicate with this person or call for the appointment?” They just shove the folder aside. We thought it would help to write ‘Deaf’ on my folder so they can lift it up instead of calling out the name so I can tell it’s for me. So that’s been a bit better lately.’ (Participant 10, Male, 45 years old)

Not all participants left health services dissatisfied and frustrated, as two participants had positive experiences at a local facility because staff agreed to remove their masks so they could lipread. Both attributed this exception to an existing awareness of Deaf culture among clinic staff:

‘The hospital knows I’m Hoh, so they know to take their mask off. There’s an employee who has a Deaf family member and taught other staff how to communicate and interact with Deaf patients.’ (Participant 11, Female, 47 years old)

South Africa’s vaccine roll-out programme began in February 2021, targeting frontline healthcare workers, followed by an age-based eligibility over the next 6 months. At the time of interviewing, 14 of 15 participants (93%) were vaccinated. Nine received their vaccine with DCCT, who arranged for an interpreter to accompany them, while three went independently with an interpreter. Among those vaccinated, 11 (79%) stated they would not have gone if DCCT had not shared information about the vaccine and arranged to have an interpreter present so they could communicate effectively and ask questions:

‘If I hadn’t had an interpreter there, what if I couldn’t see my name called and missed my appointment? What if I said yes but didn’t know which vaccine I’m getting? What if I had an allergic reaction because they didn’t ask the right questions, or I didn’t understand correctly so gave the wrong answer?’ (Participant 15, Male, 45 years old)

Two participants were vaccinated without an interpreter present. One explained the difficulty of navigating the process without an interpreter:

‘There were no interpreters at the vaccine sites but because I wanted to protect myself, I just went. There was no one to explain the process, it was very difficult for me to understand. Communication would have been smoother with an interpreter … I felt stressed and under pressure. I tried written communication, but it wasn’t easy.’ (Participant 5, Male, 41 years old)

## Discussion

This study focused on the ways in which COVID-19 policies impacted Deaf persons’ access to COVID-19-related information, to communicate, and access quality health and social services. The inaccessibility of health services for Deaf patients and the consequences of providers’ negative attitudes and discrimination towards PWDs are longstanding, global issues (Masuku, Moroe & Van Der Merwe 2021). Findings from this study and several others provide evidence that the existing discriminatory practices and communication barriers in the health system worsened during the pandemic (Hlongwane et al. 2022; Ned et al. 2020). The exclusion of interpreters as an essential service, imposition of mask mandates, and the consequential reliance on written communication not only compromised the quality-of-care participants received, but further infringed on their right to receive health information in their preferred language, as granted in the *South African Health Act (61 of 2003)*, as well as their constitutional right to access quality health care (Haricharan et al. 2013).

This study also found a few positive examples where accommodations, such as allowing an interpreter or removal of a mask, were granted due to an existing relationship and a common understanding of the needs of Deaf people among staff. This suggests that greater efforts to prioritise education, communication and trust to foster relationships can improve the cultural competency of providers and empower Deaf patients in the health system.

The lack of accessible, timely COVID-19-related information was a primary concern. Although respondents were well-informed of preventive measures and regulations at the time of interviews, they had little to no access to reliable information in SASL for the first several months of the pandemic due to the closure of DCCT, shelter-in-place orders, and insufficient interpreted news. The limitation of information available to Deaf people is not a new phenomenon (Chininthorn et al. 2016); however, its consequences are magnified during a pandemic, where information is developing rapidly and a lack of access to accurate information can be life-threatening. Several participants in this study reported not adhering to protective measures and lockdown mandates because of an information deficit, thus increasing both their risk of infection and the potential legal consequences of not following mandated regulations. As a grim example of the latter, two Deaf Ugandans were shot at by authorities for being outside during the mandated curfew; however, they had no access to information regarding the measures in place and thus, were unaware of the curfew (Brennan 2020). These findings highlight how SASL, like other minoritised languages, was disregarded in crisis communications and as such, severely threatened Deaf persons’ health and safety.

Linguistic isolation during the pandemic has been reported globally, not only by Deaf people (Panko et al. 2021; Swanwick et al. 2020) but also by many other linguistic minorities (Ndlovu & Dube 2021). Linguistic minorities reported more misunderstandings of COVID-19 preventive behaviours and greater reliance on informal information sources, such as social media or community leaders (Torensma et al. 2021). The provision of public health messaging in one’s native language, from trusted sources, that is both linguistically and culturally accurate increases the support for and adoption of recommended actions (Di Carlo et al. 2022). In Eliaz et al. (2022), language concordance during contact tracing calls was associated with 20% greater odds of COVID-19 testing and 53% greater odds of accessing support services. This was substantiated by participants in this study, who reported improved adherence to COVID-19 regulations after receiving information in SASL from DCCT or their Deaf peers. Thus, the failure to recognise the Deaf community as a linguistic minority and promote information in SASL put those at risk of the adverse effects of an information deficit during the COVID-19 pandemic.

The findings also illustrated how this information isolation was replicated within the home, which was a challenge largely unique to Deaf people, as many lived with hearing family members who were not proficient in SASL, and thus had limited capacity to share complex information. Similarities can be found in Adigun, Vivekanantharase, and Obosu (2021), whose Deaf respondents were unable to rely on their hearing family members as sources of COVID-19 information. However, hearing families frequently shared COVID-19-related information with one another, which was associated with positive family well-being and greater adherence to preventive behaviours (Wong et al. 2020). Commonly referred to as the *dinner table syndrome* (Meek 2020), the exclusion from daily conversations is not a new phenomenon but with lockdown regulations mandating people remain home, Deaf people were not only deprived of information but also of sign-deaf spaces, which are ‘networks of social relationships that function to create spaces of shared language, familiarity, sociability and communality in an often-hostile hearing world’ (Heap 2006). This loss of social cohesion, where collective gathering is the norm, diminished the ability of Deaf persons to communicate and congregate in a culturally normative way, thus limiting their ability to express part of their Deaf identity and impacting their mental health.

Our findings echo results from other studies (Silva et al. 2020; Tomasuolo et al. 2021) demonstrating the notable work of Deaf organisations globally to address the lack of support and pandemic information from governments. Deaf Community of Cape Town acted as a communication platform, source of COVID-19 information, and support system throughout the pandemic that was essential for the Deaf community. Participants expressed that COVID-19-related information provided by DCCT was largely the only information in SASL many had access to. Deaf Community of Cape Town’s swift action and creative utilisation of social media to distribute information to the Deaf community speak to their resilience.

Further, the high proportion of vaccinated participants can likely be attributed to DCCT, who disseminated accurate, timely vaccine information and provided an interpreter at the vaccine site. Despite recommendations from the WHO to consider PWDs as Stage II priority cases for vaccination, they were not prioritised in South Africa’s vaccination strategy. Among a sample of 402 PWDs in South Africa who completed an online survey between July and August 2021, only 10% had received a vaccine, although 75% were willing to be vaccinated (Hart et al. 2021). The proportion of vaccinated participants in this study, 93% (14 of 15 participants), also far surpassed the 25% national rate at the time of interviews (October 2021). The importance of prioritising vaccination for the Deaf community, as one of the many vulnerable groups, and the effectiveness of facilitating vaccination through trusted community organisations like DCCT, are illustrated here. High levels of vaccination among the Deaf community and among those who provide services to the Deaf community would enable easier communication without the barriers of masking and other precautions.

In South Africa, various governmental responses bypassed the Deaf community, largely due to the exclusion of Deaf representatives and a lack of consultation with Deaf organisations during the design of the pandemic response. For example, although substantial efforts and resources were put into contact tracing, there were no guidelines to inform tracers on how to contact and communicate with a Deaf or Hoh case, preventing them from receiving critical information regarding COVID-19 risk, testing and isolation and available resources, such as food parcels or isolation facilities. Many other South African DPOs expressed a lack of support and recognition from government during the pandemic, which Hlongwane et al. (2022) deemed as unsurprising, given that PWDs and disability-inclusive approaches are not mentioned in the national *Disaster Management Act 57 of 2002*.

Additionally, despite NGOs and community networks coming together to support the most vulnerable throughout the pandemic, DCCT, like other DPOs, was overlooked in these networks. In the Western Cape, over 170 mutual aid initiatives were developed within the first 2 months of the pandemic and provided support and resources for thousands of people (Van Ryneveld, Whyle & Brady 2022). However, these community networks neglected to partner with or communicate with DCCT or any other DPO, excluding Deaf persons and other PWDs from invaluable resources and support, likely creating further access gaps. These examples demonstrate the missed opportunities for collaboration and inclusion that occurred not only with respect to government policy omissions but also on community level and in civil society networks.

### Implications

There is little research into the impacts of the COVID-19 pandemic on Deaf people, especially in LMICs. This study was unique in that it examined the ways in which the pandemic response measures impacted Deaf people within the Deaf community, rather than only the Deaf experience within the hearing world. These findings offer meaningful and important contributions to the body of literature on the Deaf experience and pandemic research. Additionally, the findings offer important policy implications for the inclusion of Deaf and PWDs in disaster planning and management, both at national and community levels. Further research on Deaf persons’ pandemic experiences is crucial to inform effective, data-driven solutions.

### Limitations

Due to COVID-19 regulations, participants were recruited because of their connection to DCCT, which may have limited the variety of experiences reported. There is an inherent limitation when conducting research across languages, especially when those languages differ in modality. The use of qualified, professional interpreters that are experienced with research, method triangulation, and rounds of consultation was employed to minimise this limitation. Additionally, DCCT was chosen due to their long-standing relationship with UCT researchers, which has created trust and commonality over the years, empowering those in the Deaf community during the research process. The small sample size means the findings are not generalisable to the wider population; however, qualitative research aims to generate in-depth, rich data, rather than generalisability, which this study provided.

## Conclusion

These findings demonstrate how the pandemic and its response measures have both exacerbated existing inequities regarding access to information, health and social services, as well as created new challenges for Deaf people. The absence of understandable COVID-19 information for linguistic minorities is a global problem and the information deficit it produces has potentially devastating consequences. The systematic exclusion of Deaf people in South Africa’s pandemic response is symptomatic of a broader omission of Deaf people’s needs from policy responses, despite numerous state commitments, such as the Constitution and the ratification of the Convention on the Rights of Persons with Disabilities (CRPD). To mitigate this, we encourage policymakers to uphold their commitments and ensure policies are inclusive for Deaf people. The recent constitutional amendment to Section 6(1) to include SASL as the country’s 12th official language creates both an opportunity and duty to ensure Deaf persons have equal access to services and information in SASL as any other.

Within the health system, there is a need to raise awareness for healthcare workers to understand the barriers that Deaf people face when accessing care and to include Deaf representatives on clinical boards and health committees. Introducing Deaf awareness into national curriculums, implementing consistent staff training on Deaf linguistic rights and creating Deaf cultural awareness events for both staff and patients are some ways in which Deaf needs can be effectively integrated into the health system.

This study also evidenced how providing information in one’s native language through trusted sources and social networks improves behaviour change outcomes. To best promote access to and acceptance of public health messaging, we suggest that local actors and policy leaders take better initiative to actively engage with Deaf organisations, such as DCCT, to collaborate on the creation and dissemination of health communications.
